# Safety and Efficacy of Suprachoroidal Injection of Triamcinolone in Treating Macular Edema Secondary to Noninfectious Uveitis

**DOI:** 10.7759/cureus.20038

**Published:** 2021-11-30

**Authors:** Junaid Hanif, Kashif Iqbal, Fauzia Perveen, Amina Arif, Rana N Iqbal, Farukh Jameel, Kashif Hanif, Ahmad Seemab, Ahmad Y Khan, Moiz Ahmed

**Affiliations:** 1 Ophthalmology, National Eye Center, Lahore, PAK; 2 Ophthalmology, Layton Rahmatulla Benevolent Trust (LRBT) Free Eye Hospital, Lahore, PAK; 3 Pharmacology, Sharif Medical College, Lahore, PAK; 4 Medicine, Sharif Medical and Dental College, Lahore, PAK; 5 Ophthalmology, Services Hospital Lahore, Lahore, PAK; 6 Medicine, Sahara Medical College, Narowal, PAK; 7 Medicine, Jinnah Postgraduate Medical Centre, Karachi, PAK; 8 Medicine and Surgery, Sindh Medical College, Karachi, PAK

**Keywords:** edema, efficacy, safety, uveitis, medical, injection

## Abstract

Introduction

One of the leading causes of blindness throughout the world is uveitis, which predominantly results in the feared complication of macular edema. We report the safety and efficacy of suprachoroidal injection of triamcinolone acetonide in the treatment of macular edema secondary to noninfectious uveitis.

Methodology

This prospective, nonrandomized interventional study was conducted at Layton Rahmatullah Benevolent Trust (LRBT) Eye Hospital, Lahore, from August 2019 till July 2020. All individuals older than 18 years, nonpregnant females with a central macular thickness of >320 µm were included. Those patients with uncontrolled diabetes, immunodeficiency, or any other disease mandating systemic corticosteroid use were excluded. All patients had a detailed ocular exam one week before the treatment, and 0.1 ml of triamcinolone acetonide 40 mg/ml was injected using a 30-G hollow needle into the suprachoroidal space. After the injection, an eye patch was applied and the patient was observed for three hours. All data were documented in a preformed proforma.

Results

A total of 30 patients were included in the study with a mean age of 38.1 ± 9.48 years. Statistically significant differences were found between central macular thickness at presentation and at one and three months of the procedure, i.e., 569.60 ± 170.396, 266.77 ± 73.127, and 208.27 ± 37.292 µm, respectively. A similar difference was observed when comparing visual acuity at baseline to visual acuity at one and three months of the procedure (p < 0.001).

Conclusion

The current study indicates that a single dose of suprachoroidal injection of triamcinolone acetonide for the treatment of macular edema secondary to uveitis is safe and efficacious. No rise in intraocular pressure (IOP) was observed during the study period. Significant improvements in central macular thickness and visual acuity as well as tolerability and safety of the treatment were seen in our study. Further larger-scale studies are needed to ascertain the long-term benefits of the suprachoroidal triamcinolone acetonide.

## Introduction

One of the leading causes of blindness throughout the world is uveitis, which predominantly results in the feared complication of macular edema, subsequently leading to vision impairment or loss. Although uveitis has many subtypes (anterior, posterior, intermediate, or panuveitis), any of these subtypes can lead to the development of macular edema and its associated morbidity. Previous reports have suggested that up to 20% of the cases of blindness are caused by complications resulting from uveitis [1,2]. 

Noninfectious uveitis and the macular edema caused by it have long been treated using corticosteroids including prednisolone, triamcinolone acetonide, and dexamethasone. These steroids can be given locally or systemically. Traditionally, the local routes of administration have been topical drops, periocular or intravitreal injection, and intravitreal implants [3]. Despite the efficacy of these steroids in treating noninfectious uveitis, the adverse events caused by these agents present a significant treatment challenge. Administered locally, corticosteroids have a propensity to cause cataract formation, glaucoma, and elevation of intraocular pressure. A longer duration of treatment is required if corticosteroids are administered systemically to treat and maintain quiescence in uveitis. This long-term treatment often results in the systemic adverse effects of corticosteroids including hyperglycemia, osteoporosis, peptic ulceration, muscle loss, and weight gain [4]. 

Recently, a novel route of administration has come under investigation for ocular drug delivery [5]. The suprachoroidal route of administration has several proposed advantages over the traditional local routes as well as the systemic route [6]. It is expected to provide high volumes of drugs to the posterior segment while limiting exposure of the anterior segment structures [7,8]. Studies on cadaveric human eyes have demonstrated that suprachoroidal injection of triamcinolone acetonide results in the concentration of the drug in the retina, choroid, and sclera, while minimum concentrations were detected in the anterior chamber, lens, and vitreous [9,10]. The suprachoroidal injection causes the drug to move through the suprachoroidal space posteriorly resulting in the observed drug concentrations [11,12]. 

Yeh et al. presented their data on the use of suprachoroidal triamcinolone acetonide injection for the treatment of macular edema secondary to uveitis [13]. They included 22 patients, and the results showed promising results in terms of decreasing central macular thickness (CMT) and improving vision [13]. Although their results were promising, the small sample size and the proprietary formulation provided a rationale for further investigation of this treatment modality. Due to the unavailability of local studies and inconsistencies in the existing literature, we planned the study to find out the safety profile of suprachoroidal injection of triamcinolone acetonide as a management option for macular edema. We reported the safety and efficacy of suprachoroidal injection of triamcinolone acetonide in the treatment of macular edema secondary to noninfectious uveitis. 

## Materials and methods

This prospective, nonrandomized interventional study was conducted at Layton Rahmatullah Benevolent Trust (LRBT) Eye Hospital, Lahore, from August 2019 till July 2020. Ethical approval was obtained from the Ethical Review Committee. All individuals older than 18 years, nonpregnant females with a central macular thickness (CMT) of >320 µm were included. Only a single eye was treated per patient. All patients were diagnosed with macular edema secondary to uveitis.

Those patients with uncontrolled diabetes, immunodeficiency, or any other disease mandating systemic corticosteroid use were excluded. Monocular patients were excluded, as were those who had active infections, suprachoroidal hemorrhage, previous vitreoretinal surgery, or previous intraocular drug injections. Patients who used a topical ophthalmic steroid use within the last month were also excluded. 

Consecutive nonprobability sampling was used to recruit patients. All patients had a detailed ocular exam one week before the treatment. This included measurement of intraocular pressure, CMT, and best-corrected visual acuity (BCVA). All patients had their pupils dilated with tropicamide (Mydriacyl, Alcon) 1% ophthalmic drops, before the examination. Slit-lamp examination was performed on Haag-Streit Bern 900, slit lamp, Swiss made. IOP was measured using the Haag-Streit applanation tonometer. Optical coherence tomography (OCT) was performed using the “NIDEK RS-3000 OCT RetinaScan Advance 2 (Gamagōri, Japan)'' to measure the CMT.

The patients were called after one week for the intervention and had a reexamination before the injection. Patients had their pupils dilated with Tropicamide (Mydriacyl, Alcon) 1% drops, and topical anesthesia was applied (Proparacaine Hydrochloride 0.5% Eye drops). Triamcinolone acetonide (0.1 ml) (Kenacort, GlaxoSmithKline, London, England) 40 mg/ml was injected using a 30G hollow needle into the suprachoroidal space. After the injection, an eye patch was applied and the patient was observed for one hour for postprocedure complications. All patients were discharged home the same day and called for follow-up at one and three months. The patients had detailed ocular examinations again at both follow-up visits. All data were documented in a preformed proforma. 

## Results

Thirty patients were included in the study with a mean age of 38.1 ± 9.48 years. Male patients were 17 (56.7%), while female patients were 13 (43.3%) in number. Left eyes were involved in 18 (60%) patients, while 12 (40%) had involvement of the right eye. Mean values of visual acuity, changes in the central macular thickness (CMT), and intraocular pressure (IOP) at presentation, one month, and three months are presented in Table 1. Statistically significant differences were found between CMT at presentation and at one and three months (p-value <0.001 and <0.001, respectively) of the procedure. CMT decreased at one and three months as shown in Table 1.

**Table 1:** Age, Central Macular Thickness (CMT), Best Corrected Visual Acuity (BCVA), and Intraocular Pressure (IOP) at Presentation, First, and Third Months of the Injection.

Characteristics	Presentation	One-Month	Third-Months
Age (in years)	38.1 ± 9.48	-	-
Central macular thickness (CMT) µm	569.60 ± 170.396	266.77 ± 73.127	208.27 ± 37.292
Best corrected visual acuity (BCVA)	0.1415 ± 0.0694	0.2493 ± 0.0847	0.4692 ± 0.1628
Intraocular pressure (IOP) mmHg	17.07 ± 2.88	17.9 ± 3.37	17.37 ± 3.29

A similar difference was observed when comparing visual acuity at baseline to visual acuity at the first and third months of the procedure (p<0.001). Intraocular pressure at baseline was not significantly different from IOP at the first and third months of the procedure (p=0.26 and p=0.636, respectively). Five patients were found to have lenticular changes, but vitreous and retinal observations could still be made properly, and no other ocular adverse events were noted in our study.

## Discussion

Our study, which included 30 patients of noninfectious uveitis who were treated with a single suprachoroidal injection of triamcinolone, showed that there was a statistically significant reduction in central macular thickness (CMT). Patients experienced a reduction of more than 50% of the initial thickness (mean CMT at presentation was 569.6±170.39 µm as compared to 266.7±73.127 µm at one month) which resulted in the resolution of macular edema of all the patients. At three months, this reduction in CMT was not only sustained but improved with a further reduction of 22% over the CMT at one month (mean CMT at three months was 208.2±37.29 µm). 

Previous studies have demonstrated that in patients suffering from macular edema, a 20% change in macular thickness is associated with improved visual acuity; this finding correlates with our results as our patients not only had a reduction in CMT but had improvement in visual acuity as well [14]. Visual acuity in our study also showed a significant improvement after the resolution of macular edema. Mean BCVA at one month was 0.2493 ± 0.0847 (p<0.001) and was further improved at three months 0.4692±0.1628 (p<0.001). This shows that an anatomical improvement after suprachoroidal injection of triamcinolone was also accompanied by a functional improvement in our study. 

Current treatment options for macular edema due to uveitis include topical as well as intravitreal corticosteroids injections and implants. Varying levels of success have been seen with these treatment modalities, but this success has also been associated with risks of raised IOP, cataract formation, and progression. These adverse events are a significant concern in patients being treated with topical or intravitreal corticosteroids with up to 17% of patients experiencing cataract progression in periocular steroid instillation and up to 66% of patients having raised IOP after intravitreal injection of steroids [15]. Long-term ocular treatment with steroids using current modes of administration has been reported to lead to an alarmingly high level of IOP lowering medications and procedures as well as cataract formation and surgery [16]. None of the patients in our study experienced a rise in IOP, with mean IOP remaining within the normal range on all measurements. Although data in particular regard are scarce, previous studies have demonstrated that suprachoroidal injections are safer in terms of rising in IOP. Adverse events that we did observe were that five patients had changes in their lens/vitreous humor, but these changes did not preclude the vision or examination of retina/macula. In another study, Tayyab et al. revealed that suprachoroidal triamcinolone acetonide significantly improved central subfield thickness (CST) at three months of procedure (p<0.00001) [17]. The study showed promising outcomes for diabetic macular edema. 

Fewer adverse events seen in our study can be considered as evidence in terms of the safety of the use of suprachoroidal triamcinolone. A small sample size, noncomparative nature, and short-term follow-up in our study preclude us from determining the long-term safety and efficacy of the treatment. Longer follow-up is required to understand whether the improvement is sustained over long periods of time or reinjection is required. Profiles of adverse events will also be more profound and clear in longer-term follow-up. If reinjections are required, they may increase the risk of developing the adverse events we discussed earlier. Comparisons with topical or intravitreal injection of corticosteroids should be the next step, which will yield a higher quality of evidence.

One of the limitations of the current study was the small and undiversified sample size. This limited the interference of our findings to a larger population. Despite the shortcomings of our study, we have demonstrated the safety and efficacy of a single dose of suprachoroidal injection of triamcinolone in treating macular edema secondary to uveitis over a third-month period. Significant improvements in CMT and visual acuity, as well as tolerability and safety of the treatment, were seen in our study. Further larger randomized controlled trials are needed to solidify the evidence in favor of suprachoroidal triamcinolone.

## Conclusions

The current study indicated that a single dose of suprachoroidal acetonide injection of triamcinolone for the treatment of macular edema secondary to uveitis is safe and effective. Significant improvements in central macular thickness and visual acuity as well as tolerability and safety of the treatment were seen in our study. Further larger-scale, randomized controlled trials are needed to ascertain the long-term benefits of the suprachoroidal triamcinolone.

## References

[1] Miserocchi E, Fogliato G, Modorati G, Bandello F (2013). Review on the worldwide epidemiology of uveitis. Eur J Ophthalmol.

[2] Lardenoye CW, van Kooij B, Rothova A (2006). Impact of macular edema on visual acuity in uveitis. Ophthalmology.

[3] Gaballa SA, Kompella UB, Elgarhy O, Alqahtani AM, Pierscionek B, Alany RG, Abdelkader H (2021). Corticosteroids in ophthalmology: drug delivery innovations, pharmacology, clinical applications, and future perspectives. Drug Deliv Transl Res.

[4] Nguyen QD, Hatef E, Kayen B (2011). A cross-sectional study of the current treatment patterns in noninfectious uveitis among specialists in the United States. Ophthalmology.

[5] Rai UD, Young SA, Thrimawithana TR, Abdelkader H, Alani AW, Pierscionek B, Alany RG (2015). The suprachoroidal pathway: a new drug delivery route to the back of the eye. Drug Discov Today.

[6] Chen M, Li X, Liu J, Han Y, Cheng L (2015). Safety and pharmacodynamics of suprachoroidal injection of triamcinolone acetonide as a controlled ocular drug release model. J Control Release.

[7] Habot-Wilner Z, Noronha G, Wykoff CC (2019). Suprachoroidally injected pharmacological agents for the treatment of chorio-retinal diseases: a targeted approach. Acta Ophthalmol.

[8] Wang JC, Eliott D (2017). Accessing the suprachoroidal space for therapeutic delivery. Int Ophthalmol Clin.

[9] Patel SR, Lin AS, Edelhauser HF, Prausnitz MR (2011). Suprachoroidal drug delivery to the back of the eye using hollow microneedles. Pharm Res.

[10] Hartman RR, Kompella UB (2018). Intravitreal, subretinal, and suprachoroidal injections: evolution of microneedles for drug delivery. J Ocul Pharmacol Ther.

[11] Olsen TW (2015). Suprachoroidal drug delivery: unique new observations. Invest Ophthalmol Vis Sci.

[12] Lampen SI, Khurana RN, Noronha G, Brown DM, Wykoff CC (2018). Suprachoroidal space alterations following delivery of triamcinolone acetonide: post-hoc analysis of the phase 1/2 HULK Study of patients with diabetic macular edema. Ophthalmic Surg Lasers Imaging Retina.

[13] Yeh S, Kurup SK, Wang RC, Foster CS, Noronha G, Nguyen QD, Do DV (2019). Suprachoroidal injection of triamcinolone acetonide, CLS-TA, for macular edema due to noninfectious uveitis: a randomized, phase 2 study (DOGWOOD). Retina.

[14] Sugar EA, Jabs DA, Altaweel MM, Lightman S, Acharya N, Vitale AT, Thorne JE (2011). Identifying a clinically meaningful threshold for change in uveitic macular edema evaluated by optical coherence tomography. Am J Ophthalmol.

[15] Karim R, Sykakis E, Lightman S, Fraser-Bell S (2013). Interventions for the treatment of uveitic macular edema: a systematic review and meta-analysis. Clin Ophthalmol.

[16] Kempen JH, Altaweel MM, Holbrook JT, Jabs DA, Louis TA, Sugar EA, Thorne JE (2011). Randomized comparison of systemic anti-inflammatory therapy versus fluocinolone acetonide implant for intermediate, posterior, and panuveitis: The Multicenter Uveitis Steroid Treatment Trial. Ophthalmology.

[17] Tayyab H, Ahmed CN, Sadiq MA (2020). Efficacy and safety of suprachoroidal triamcinolone acetonide in cases of resistant diabetic macular edema. Pak J Med Sci.

